# Infant mortality due to perinatal causes in Brazil: trends, regional patterns and possible interventions

**DOI:** 10.1590/S1516-31802001000100009

**Published:** 2001-01-02

**Authors:** Cesar Gomes Victora, Fernando Celso Barros

**Keywords:** Infant mortality, Perinatal causes, Ante-natal care, Delivery care, Preventive care, Mortalidade infantil, Causas perinatais, Pré-natal, Parto, Prevenção

## Abstract

**CONTEXT:**

Brazilian infant and child mortality levels are not compatible with the country's economic potential. In this paper, we provide a description of levels and trends in infant mortality due to perinatal causes and malformations and assess the likely impact of changing intermediate-level determinants, many of which are amenable to direct interventions through the health or related sectors.

**TYPE OF STUDY:**

Review paper.

**METHODS:**

Two main sources of mortality data were used: indirect mortality estimates based on censuses and surveys, and rates based on registered deaths. The latter were corrected for under-registration. Combination of the two sources of data allowed the estimation of cause-specific mortality rates. Data on current coverage of preventive and curative interventions were mostly obtained from the 1996 Demographic and Health Survey. Other national household surveys and Ministry of Health Statistics were also used. A thorough review of the Brazilian literature on levels, trends and determinants of infant mortality led to the identification of a large number of papers and books. These provided the background for the analyses of risk factors and potential interventions.

**RESULTS:**

The indirect infant mortality rate estimate for 1995-97 is of 37.5 deaths per thousand live births, about six times higher than in the lowest mortality countries in the world. Perinatal causes account for 57% of all infant deaths, and congenital malformations are responsible for 11.2% of these deaths. Mortality levels are highest in the Northeast and North, and lowest in the South and Southeast; the Center-West falls in between. Since surveys of the North region do not cover rural areas, mortality for this region may be underestimated.

**CONCLUSIONS:**

A first priority for the further reduction in infant mortality in Brazil is to improve equality among regions, since the North and Northeast, and particularly rural areas, still show very high death rates. Further reductions in infant mortality will largely depend on decreasing deaths due to perinatal causes. Improvements in the coverage and particularly in the quality of antenatal and delivery care are urgently needed. Another intervention with a potential important impact on infant mortality is the promotion of family planning. Improving birth weight might lead to an 8% reduction in infant mortality but the efficacy of available interventions is low.

## RESUMO

**CONTEXTO:**

Os coeficientes brasileiros de mortalidade infantil e de crianças abaixo de 5 anos de idade não são compatíveis com o potencial econômico do país. Neste artigo descrevemos os níveis e tendências da mortalidade infantil por causas perinatais e malformações, e avaliamos o possível impacto de modificações de seus determinantes intermediários, através de intervenções diretas do setor saúde ou setores relacionados.

**MÉTODOS:**

Duas fontes de dados de mortalidade foram utilizadas: estimativas indiretas de mortalidade baseadas em recenseamentos e inquéritos e coeficientes calculados a partir de registros de óbitos. Esses últimos foram corrigidos para sub-registros. A combinação dessas duas fontes permitiu a estimativa de coeficientes de mortalidade por causas específicas. Dados sobre a cobertura de atenção de saúde foram obtidos da Pesquisa Nacional de Demografia e Saúde de 1996, assim como de estatísticas do Ministério da Saúde. Realizamos uma ampla revisão da literatura brasileira sobre níveis, tendências e determinantes da mortalidade infantil. As informações contidas em grande número de artigos e livros possibilitaram a análise de fatores de risco e possíveis intervenções.

**TIPO DE ESTUDO:**

Artigo de revisão.

**RESULTADOS:**

A estimativa indireta do coeficiente de mortalidade infantil para 1995-97 é de 37.5 mortes por 1.000 nascidos vivos, cerca de 6 vezes mais alta do que a dos países do mundo com os coeficientes mais baixos. Os coeficientes de mortalidade mais elevados são encontrados no Norte-Nordeste, e os mais baixos no Sul-Sudeste; o Centro-Oeste se situa em uma posição intermediária. Uma vez que os inquéritos na região Norte não incluem áreas rurais, os coeficientes de mortalidade infantil dessa região podem estar subestimados. Para todo o país, as causas perinatais são responsáveis por 57% de todas as mortes infantis, e as malformações congênitas são responsáveis por 11.2% dessas mortes.

**CONCLUSÕES:**

A primeira prioridade para uma maior redução da mortalidade infantil no Brasil é melhorar a eqüidade entre as regiões, uma vez que o Norte e o Nordeste e, particularmente, as áreas rurais, ainda apresentam coeficientes muito elevados. Reduções subseqüentes da mortalidade infantil vão depender, em grande parte, da redução das mortes devidas a causas perinatais. Melhorias na cobertura e, especialmente, na qualidade da atenção ao pré-natal e ao parto, são necessidades urgentes. Outra intervenção, com um possível impacto importante sobre a mortalidade infantil, é a promoção do planejamento familiar. A melhoria do peso ao nascer poderia levar a uma redução de 8% na mortalidade infantil, mas a eficácia das intervenções disponíveis é muito baixa.

## INTRODUCTION

Infant and child mortality levels for Brazil are not compatible with the country's economic potential. Brazil ranks in 85th place among the world's 192 nations ranked according to under-five mortality.^1^ Although progress has been made in recent years, further interventions are clearly necessary for improving the survival of our children.

The conceptual framework guiding the present analysis is based on that proposed originally by Mosley and Chen^2^ and further developed by others.^3^ The diseases leading to the fatal outcome - for example perinatal conditions - constitute the immediate (or proximate) causes of death. Their occurrence is ultimately determined by macro-level social, economic and cultural factors - such as income, education and land tenure - which constitute the distal determinants of mortality. These factors influence the occurrence of the immediate causes of death by affecting intermediate-level determinants, either by increasing exposure to risk factors (for example, poor nutrition during pregnancy, etc) or by decreasing access to protective factors (for example, antenatal and delivery care, etc).

In this review paper, we provide a description of levels and trends in infant mortality due to perinatal causes and malformations and assess the likely impact of changing intermediate-level determinants, many of which are amenable to direct interventions through the health or related sectors. The importance of distal determinants cannot be over-emphasized. These are historically responsible for the high levels of mortality in the country and for the sharp regional inequalities, but being less amenable to direct, short-term interventions, they will not be addressed in this review. In the evaluation of risk factors for mortality, special care was taken to rely on studies in which the confounding effects of socioeconomic status were controlled for. This will help identify interventions that are causally related to mortality, rather than factors whose association with mortality is spurious, resulting from their higher frequency in poor households.

We start by reviewing available studies of the proximate and indirect determinants of infant mortality due to perinatal causes in Brazil. We then analyze data sources on infant mortality, and describe recent time trends and geographical distribution according to the five regions of the country (North, Northeast, Southeast, South and Center-West).

Next, the effectiveness of available interventions against perinatal causes is reviewed, and existing data on the current coverage of these interventions are presented. Based on these data and on informed judgement, the relative effectiveness of the various interventions will be judged. Finally, the feasibility of increasing the coverage of each intervention will be addressed, and specific recommendations for action will be made.

## METHODS

### Mortality data sources and methods

Two main sources of infant mortality data were used in this review: indirect mortality estimates based on demographic censuses and surveys, and rates based on registered deaths. Each of these sources has advantages and disadvantages, which are outlined below.

### Indirect estimates from census and surveys.

The methods developed by Brass and Trussel^4^ allow the estimation of infant and child mortality rates from census and survey information. Women of reproductive age are requested to answer questions on the number of children ever born and of children surviving, as well as on births in the preceding 12 months. Such information is modeled to provide mortality estimates for different ages in childhood.

A recent publication from the Brazilian Institute for Demography and Statistics (Instituto Brasileiro de Demografia e Estatística – IBGE) and the Ministry of Health^5^ provides regional mortality trends based on the 1980 and 1991 Demographic Censuses, and on the National Household Surveys (Pesquisas Nacionais por Amostragem Domiciliar - PNAD) of 1992 through 1996. In the present document, results from this publication as well as special tabulations provided by C. Simões were used to describe regional mortality levels and trends between 1980 and 1998.

Results from these different censuses and surveys were smoothed using a logistic function and extrapolated to provide mortality estimates up to 1998. A possible caveat to be taken into account when interpreting these results is that due to the curve-fitting procedure, short-term mortality fluctuations are not apparent and relatively smooth curves are produced.

The estimates derived by IBGE are close to those obtained through the 1996 National Demographic and Health Survey, part of the global DHS initiative. This survey^6^ - hereafter referred to as 1996 DHS - provided regional estimates of infant mortality for 1991, of 43 (North - urban areas only), 74 (Northeast), 38 (Southeast), 25 (South) and 39 (Center-West) deaths per 1,000 live births. For the same year, the rates estimated by IBGE were respectively, 42, 71, 32, 26 and 30. The national IMR for 1991 was equal to 48 according to DHS and 45 according to IBGE.

*Official mortality statistics.* Information on all registered deaths in Brazil are collected by local registrars and forwarded to the State Secretariats of Health, where these are compiled and sent to the Ministry of Health in Brasília. The Mortality Information System (Sistema de Informações de Mortalidade – SIM) is available online^7^ and provides mortality information up to the end of 1997. The following ICD-BR codes were used to identify deaths due to peri-natal causes: code 045 for 1979-95 (9^th^ revision of the ICD) and codes 092-096 for 1996-97 (10^th^ revision). For congenital malformations, the respective ICD codes were 044 and 097-099.

The main problem affecting official statistics is the under-registration of deaths. The above-cited publication by Simões^5^ estimates that, in 1996, the following percentages of all deaths were not registered: North (41.3%), Northeast (44.9%), Southeast (1.7%), South (1.8%) and Center-West (17.4%). The national rate was 19.1%. For infant deaths, the corresponding under-registration estimates were 52.2%, 66.7%, 6.5%, 13.6% and 23.9%, the national rate being equal to 43.7%. It is likely that most deaths that are not registered occur in the rural areas of the North and Northeast, and causes of death that are particularly common in these areas - for example, infectious diseases - may be underestimated.

A possible problem with the IBGE estimates is that the late-1990s mortality levels were based on projections from early-1990s data. Since under-registration is estimated by dividing registered deaths by the expected number of deaths based on indirect estimates, an apparently high rate of under-registration will be observed if mortality rates are falling faster than predicted by the IBGE estimates. However, there is no way to assess the magnitude of this problem until the year 2000 demographic census provides new estimates of mortality levels in the late 1990's.

Under-registration of births can also distort infant mortality estimates. Although the Ministry of Health computerized birth records system (SINASC - Sistema de Informaçoes sobre Nascimentos) has improved substantially in recent years, its coverage is still low in areas with many home deliveries. Under-registration of births can be overcome by using census information on the number of children under one year of age to estimate the number of live births. The estimates calculated by Simões^5^ were used.

Information on registered deaths was used to calculate proportionate infant mortality ratios (proportion of infant deaths relative to all deaths at all ages) and proportionate infant mortality due to perinatal causes and malformations. Since all of these proportions use as denominators the numbers of registered deaths, they are less affected by under-registration (since this would also affect their denominator).

A further problem that may affect cause-specific analyses is the high proportion of deaths attributed to ill-defined causes (including deaths without medical assistance and those attributed to non-specific signs and symptoms). This proportion is particularly high in the North and Northeast regions, 16.0% and 25.5% in 1995-7, respectively. These deaths were excluded from the denominator for the calculation of cause-specific proportionate mortality.

To estimate infant mortality rates due to perinatal causes (per thousand live births), the proportions of registered deaths due to perinatal causes were multiplied by the indirect estimate of the infant mortality rate. These cause-specific rates were then multiplied by the estimated number of live births to calculate the annual absolute number of deaths by cause for 1995-7.

### Literature review of determinants of infant mortality

A systematic review of the scientific literature from 1970 to 1999 was performed in three major databases, MEDLINE (National Library of Medicine, USA), POPLINE (Johns Hopkins University, USA) and LILACS (Latin American Literature on Health Sciences, Regional Library of Medicine, Brazil). While MEDLINE is restricted to articles published in indexed journals, both POPLINE and LILACS also include unpublished research such as conference proceedings, book chapters and research reports.

Over 300 papers were identified in these three databases. A short list of possibly relevant papers was drawn and copies of these papers were obtained for review. Among these papers, we selected high-quality studies in which under-reporting of deaths was avoided, either by active surveillance of deaths or because the study was carried out in an area with low rates of under-registration. Whenever the high-quality Brazilian literature was restricted, international sources were used, preferably meta-analyses when available. This list was complemented by personal contacts with relevant institutions including PAHO, UNICEF, IBGE, the Ministry of Health and BEMFAM (the institution in charge of the DHS surveys in Brazil). The results of this literature review are presented below in the following sections.

### Infant mortality levels and trends

The time frame for the present analyses was defined on the basis of data availability. Registry-based information on the number and causes of death is available up to 1997, and projections of indirect infant mortality estimates are available up to 1998, based on actual data from the early 1990s. Since most of the information on underlying determinants of mortality came from the two DHS surveys (1986^8^ and 1996^6^), mortality rates were averaged for two three-year periods (1985-7 and 1995-7) and the analyses were based on a comparison between these periods. The use of three-year averages resulted in mortality measures that were less affected by annual fluctuations.

Table 1 shows that national infant mortality levels, according to indirect estimates, fell by about 40% between 1985-7 and 1995-7. Data from registered death certificates confirmed the national decline in infant mortality, from 128,745 annual deaths in 1985-7, to 75,990 a decade later, a 41.0% reduction. Infant mortality has been declining in Brazil for several decades, but progress has been slower than for most countries in the Americas. According to PAHO,^9^ Brazil is in the group of countries with an IMR decline of 60-69% between 1950-5 and 1990-5. Of the countries studied, 23 showed faster declines than Brazil, and only nine had slower rates of decline.

**Table 1:** Infant mortality statistics for the Brazilian regions, 1985-7 and 1995-7: indirect estimates based on censuses and surveys, and vital registration statistics

Year		Region					Brazil
		North	Northeast	Southeast	South	Center-West	
**Indirect IMR estimate**	1985-7	58.9	90.6	44.6	37.7	40.8	62.4
	1995-7	36.2	60.5	25.9	22.8	25.8	37.5
% reduction		38.6%	33.2%	42.1%	39.5%	36.8%	39.8%
**Percent of infant over all registered deaths**	1985-7	26.6%	23.4%	12.9%	11.7%	14.1%	16.1%
	1995-7	15.7%	11.6%	7.0%	6.3%	9.5%	8.4%
% reduction		41.0%	50.4%	46.0%	46.7%	32.9%	47.6%
**Percent of neonatal over all infant deaths**	1985-7	40.6%	35.1%	55.9%	51.9%	52.4%	46.4%
	1995-7	60.1%	50.5%	65.0%	59.2%	77.6%	59.2%
% increase		48.0%	43.9%	16.3%	14.1%	48.1%	27.6%
**Percent of infant deaths according to cause** 1							
Perinatal causes	1985-7	42.1%	42.9%	48.9%	46.4%	48.7%	46.5%
	1995-7	61.4%	53.9%	58.8%	53.0%	56.7%	56.8%
Malformations	1985-7	4.9%	3.6%	8.1%	11.4%	8.6%	7.1%
	1995-7	8.5%	7.3%	12.2%	16.2%	13.4%	11.2%
**Estimated cause-specific IMR**							
Perinatal causes	1985-7	24.8	38.9	21.8	17.5	19.9	29.0
	1995-7	22.2	32.6	15.2	12.1	14.6	21.3
Malformations	1985-7	2.9	3.3	3.6	4.3	3.5	4.4
	1995-7	3.1	4.4	3.2	3.7	3.4	4.2

1 *Expressed as percentages of all deaths with a valid cause (excluding ill-defined causes).*

All regions of the country showed declines, ranging from 33.2% in the Northeast to 42.1% in the Southeast (Table 1). Strong regional differences persisted, with 1995-7 IMR levels in the Northeast being almost three times higher than in the South, Southeast and Center-West.

Multiplying the indirectly-estimated infant mortality rates by the number of births in each region provides an estimate of 12,344 annual deaths in the North region for 1995-7; 68,171 for the Northeast; 33,225 for the Southeast; 10,497 for the South; 5,992 for the Center-West; and 129,305 for Brazil as a whole.

Proportionate mortality statistics, based on vital registration, also showed important progress (Table 1). When health conditions improve, infant mortality tends to fall faster than adult mortality. Infant deaths accounted for 16.1% of all deaths in 1985-7, declining to 8.4% in 1995-7. The reductions ranged from 32.9% in the Center-West to 50.4% in the Northeast. Again, regional inequities were marked, with the lowest mortality levels observed in the South/Southeast and the highest in the North/Northeast.

When infant mortality decreases, post-neonatal mortality (deaths occurring between 28 and 364 days of age) tends to fall faster than neonatal mortality (deaths from birth to 27 days). Therefore, as Table 1 shows, the proportion of infant deaths occurring in the neonatal period increased nationally from 46.4% to 59.2%. Again, the increase was observed in all regions but mostly in the North, and regional inequities were similar to those observed for the previous indicators. Although with high initial IMR levels post-neonatal mortality tends to fall faster than neonatal mortality, when a low IMR is reached this tendency may revert. This is because a very low level of post-neonatal mortality has already been reached and greater progress can then be made against neonatal deaths. This may be the case for the South, which showed the lowest level of reduction.

These observations are also consistent with those on proportionate mortality due to peri-natal causes and malformations and with the estimated IMR's due to perinatal causes and malformations, also shown in Table 1. The latter estimate was obtained by multiplying the indirect IMR by the cause-specific proportions of registered deaths, and should be interpreted as the number of deaths per thousand live births.

The leading cause of registered infant deaths in Brazil is perinatal conditions, which accounted for 46.5% of infant mortality in 1985-7 and 56.8% in 1995-7. This relative increase in proportionate mortality was observed for all regions, but the estimated national IMR due to perinatal causes was reduced from 29.0 to 21.3 per thousand. The rates are highest in the North/Northeast and lowest in the South/Southeast.

The second leading cause of registered infant deaths in 1995-7 were malformations, which overtook diarrhea and respiratory infections during the decade. These deaths are remarkably hard to prevent so that their relative weight tends to increase when mortality falls: for Brazil as a whole, the increase was from 7.1% to 11.2% of all infant deaths in the decade under study. The national IMR due to malformations remained stable: 4.4 and 4.2 per thousand. The apparent increase in the Northeast may also be attributed to improved certification. The fact that malformations show little variability from one region to another is a good indicator of data consistency.

The third cause of infant deaths in the country was acute respiratory infections, mostly pneumonia, followed by diarrhea and other infections (data not shown).

### Potential interventions against perinatal-cause mortality

Table 2 lists the major potential interventions against infant deaths due to perinatal causes and malformations. Available indicators of the current coverage of these interventions, by region, are shown in Table 3.

**Table 2:** Number of infant deaths due to main causes and possible interventions addressing these causes. Brazil, 1995-7

Proximate cause of death	Percent (annual number) of infant deaths, 1995-7	Potential interventions	Availability of population-based data at the regional level
Perinatal causes	56.8% (73,641)	Improving antenatal care (including tetanus vaccination)	Utilization indicators: lack of antenatal care; antenatal care starting in first trimester; number of antenatal attendances Quality indicator: tetanus toxoid vaccination
		Improving delivery practices and neonatal care	Utilization indicators: hospital deliveries Quality indicator: deliveries by doctors, cesarean section rate
		Improving maternal nutrition	Indicators: maternal height, maternal body mass index
		Preventing low birth weight	Indicator: low birth weight (hospital deliveries)
		Reducing maternal smoking	No indicator available
		Family planning	Indicators: adolescent pregnancies, short birth intervals
Congenital malformations	11.4% (14,487)	Improving maternal folate intake	Not available
		Antenatal syphilis treatment	Not available
		Rubella vaccination	Not available
		Alcohol avoidance	Not available

**Table 3:** Distribution of indicators relevant to possible interventions against infant mortality, by region. Brazil, circa 1996

Intervention and relevant indicators	Year and source	Region					Brazil
		North	North East	South East	South	Right-West	
**Antenal Care** Did not attend antenatal care	1996^a^	17.1%	25.2%	6.1%	4.9%	7.0%	13.2%
Antenatal care starting first trimester	1996^a^	55.7%	51.9%	74.2%	79.7%	71.7%	66.0%
Median number of antenatal attendances	1996^a^	6.3	6.4	8.1	8.3	7.6	7.4
Received 2 or more doses of tetanus toxoid	1996^a^	51.0%	49.6%	38.2%	47.1%	53.7%	45.3%
Less than 5 antenatal attendances^b^	1996^a^	44.5%	49.3%	20.8%	18.0%	24.7%	31.9%
**Delivery practices** Hospital deliveries	1996^a^	81.9%	83.4%	97.0%	97.4%	97.1%	91.5%
Deliveries by doctors	1996^a^	55.1%	57.4%	92.7%	87.6%	92.0%	77.6%
Deliveries by skilled attendants	1996^a^	75.0%	76.3%	96.1%	93.1%	96.4%	87.7%
C-section rate	1996^a^	25.5%	20.4%	47.2%	44.6%	49.1%	36.4%
**Maternal nutrition** Mean maternal height (cm)	1996^a^	154.4	154.7	157.5	157.9	156.6	156.3
Mean maternal body mass index (kg/m2)	1996^a^	23.0	23.4	24.5	24.8	23.6	24.0
Maternal BMI < 18.5 kg/m^2^	1996	5.9%	7.1%	6.6%	2.7%	8.1%	6.3%
**Birthweight** Reported prevalence of birthweight <2500 g	1996^a^	7.4%	7.4%	8.9%	7.6%	9.1%	8.1%
Birthweight information not available	1996^a^	14.9%	21.2%	5.5%	2.4%	5.7%	5.7%
Hospital birthweights <2500 g	1997c	6.4%	7.0%	8.7%	7.9%	7.1%	7.8%
Corrected low birthweight estimate^d^	1996^a^	8.5%	9.0%	9.2%	7.8%	9.4%	8.5%
**Family planning** Ever pregnant adolescents (15-19 years)	1996^a^	23.5%	20.6%	16.2%	16.2%	17.0%	18.1%
Birth interval less than 24 mos	1996^a^	33.3%	37.7%	25.6%	18.1%	22.4%	29.2%
Contraceptive use rate among married women	1996^a^	72.3%	68.2%	79.5%	80.3%	84.5%	76.7%
Total fertility rate	1996^a^	2.7	3.1	2.2	2.3	2.3	2.5

a *Source: National Demographic and Health Survey 1996 (North region: urban areas only);*

b *Calculated from the original figures by adding all mothers with no attendances, those with 1-3 attendances and one third of those with 4-6 attendances. National Birth Registration System (SINASC), Ministry of Health.; Based on 1996 DHS results but assuming that LBW prevalence was 15% when mother was not able to provide this information.*

Perinatal causes are responsible for 56.8% of all infant deaths in Brazil. This estimate does not include stillbirths, since infant mortality rates are restricted to live born children. The Northeast has the highest rates (Table 1), and there has been little progress in the last decade, whereas most other causes of infant deaths decreased sharply. Success in reducing infant mortality in Brazil will therefore be largely based on an effective impact against perinatal deaths.

Of all perinatal-cause infant deaths in 1996-97, 60.7% were due to respiratory and cardiovascular conditions specific to the perinatal period, 8.4% were due to problems affecting fetal growth and/or duration of pregnancy, 6.9% due to perinatal problems related to pregnancy complications, 0.3% due to birth trauma and 23.7% due to the remaining problems originated in the perinatal period. It was not possible to further subdivide the respiratory and cardiovascular conditions for those years, but in a study conducted by the Ministry of Health for a previous year (1985), the 61.4% from respiratory and cardiovascular causes were divided in the following way: respiratory distress syndrome (21.0%), hypoxia or anoxia (11.7%) and other respiratory conditions (28.7).^10^

The following interventions are potentially effective for reducing mortality due to perinatal causes.

*Improving antenatal care*. Adequate antenatal care (ANC) can reduce mortality by detecting and treating maternal diseases (for example, syphilis, diabetes, hypertension, HIV/AIDS, and other infections), improving maternal nutrition, vaccinating against tetanus and providing health advice on smoking and drinking. This would reduce perinatal deaths due to preterm delivery, low birth weight, respiratory distress syndrome and maternal conditions.

Appropriate ANC requires that such services be accessible to pregnant women (in terms of geographical location and opening hours), affordable, and of adequate quality. Available indicators for ANC utilization in the Brazilian regions include the proportion of pregnant women who did not attend ANC, the proportion starting ANC in the first trimester of pregnancy, and the mean level of ANC attendance. The only proxy indicator available on the ANC quality is the proportion of women who received two or more immunizations against tetanus during pregnancy. Like most indicators in Table 3, these are based on the 1996 DHS^6^ and refer to children born in the five years preceding the survey.

Table 3 shows that 13.2% of Brazilian mothers failed to attend any ANC sessions, ranging from 4.9% in the South to 25.2% in the Northeast. Ideally, ANC should start in the beginning of pregnancy to allow the detection and early treatment of complications. Two thirds of Brazilian mothers who attended ANC had a first consultation within the first trimester, ranging from about 80% in the South to just over 50% in the Northeast. The mean number of ANC attendances was adequate (7.4) for the country as a whole, ranging from 8.3 in the South to 6.3 in the North and 6.4 in the Northeast.

Regarding the quality of antenatal care, about 45% of the women received two or more doses of tetanus vaccine during pregnancy. This proportion was slightly lower in the Southeast (38%) and was close to 50% in the other four regions. A further 13.2% of Brazilian mothers received a single dose. Other indicators of ANC quality are not available on a regional basis, but reports from different parts of the country suggest that poor quality is probably a greater problem than low utilization.^11^ A study from Southern Brazil^12^ showed that ANC quality was particularly poor for women with high gestational risk levels, who did not have access to private physicians nor to private health insurance.

Five studies provided quantitative information on the impact of antenatal care on child survival, controlling for confounders.^13-17^ Such adjustment is essential since mothers with fewer attendances also tend to present other social and biological risk factors. Most studies investigated early neonatal mortality - rather than mortality due to perinatal causes - but these are largely overlapping. These studies were carried out in different settings, at different times, and used different ANC categories for presenting results, but all showed a protective effect of antenatal care. Taking together the results of the three better-designed studies^13-15^ mothers with fewer than five attendances were at about 2.5 times greater risk than those with 5 or more attendances.

Assuming that the percentages of mothers with fewer than five ANC attendances could be reduced by half (from 31.9% to 16.0%), one would theoretically prevent 16.2% of all peri-natal-cause deaths. These calculations have to be interpreted with due caution due to the many underlying assumptions, but they do suggest that improving antenatal care could have a substantial impact on mortality. Existing data are not sufficient to estimate the likely impact of improving the quality of antenatal care.

*Improving delivery practices and neonatal care.* Adequate delivery care can reduce deaths due to anoxia, hypoxia and other respiratory conditions, and decrease neonatal infections, including tetanus. Appropriate care of neonates can ensure the survival of preterm and low birth weight babies that would otherwise die. Appropriate respiratory care can substantially decrease the mortality due to respiratory distress syndrome and other respiratory conditions.

Table 3 shows that over 90% of all deliveries in Brazil take place in a hospital, ranging from 81.9% in the North to 97.4% in the South. Skilled health workers - including doctors or nurses - are in charge of 87.7% of all deliveries, from 75% in the North to 96.4% in the Center-West. The sharp regional inequities observed for ANC are therefore also present for delivery care. We were unable to obtain indicators of neonatal care on a national or regional basis.

Since most Brazilian studies on neonatal survival are hospital-based, only one study provided information on mortality levels for babies delivered at home and in a hospital.^13^ This was carried out in a rural Northeast community and showed an adjusted relative risk of 1.2 (0.5-2.8) for neonatal mortality among home-delivered, compared to hospital-delivered, non-referred babies. After controlling for the existence of complications, babies who were planned to be delivered at home but had to be referred to a hospital presented a relative risk of 0.3 (0.1-1.4) of neonatal death. This reduction in risk is suggestive but not statistically significant.

The cesarean section rate in Brazil is one of the highest in the world, accounting for 36.4% of all births; the rate is lowest in the Northeast (20.4%) and North (25.5%) but represents almost half of all births in the Southeast, South and Center-West. According to the 1996 DHS, most births (52.1%) in São Paulo State are through a cesarean section.

Whereas improving delivery and neonatal care is very likely to reduce mortality, the situation for cesarean sections is more complex. Very low levels of cesarean sections increase mortality by exposing babies to the risk of birth trauma and anoxia or hypoxia. On the other hand, high cesarean rates indicate that many of these operations are elective. Errors in the calculation of gestational age can lead to preterm delivery.^18,19^

WHO recommends that not more than 15% of all deliveries should be done through a cesarean section.^20^ Reducing cesarean section rates is a very difficult task because it involves different sectors of society.^21^ Doctors used to be paid more for a cesarean section than for a vaginal delivery; although this is no longer the case, this may have been the factor that started the sharp increase in operations. Even if payment is the same, a cesarean section delivery takes less than one hour, while a vaginal delivery can take many hours; this is another powerful incentive. Mothers have been taught that cesarean sections are painless and carry no greater risk than vaginal delivery; high-income mothers have the highest cesarean section rates, and low-income mothers associate cesarean sections with the optimal medical care to which they also aspire, so they often attempt to persuade doctors to operate on them.^22^ Finally, since until recently sterilization was illegal in Brazil, mothers often requested doctors to do a cesarean section in order to carry out a simultaneous “hidden” tubal ligation;^18^ since social security did not cover this procedure, the family made an additional payment to the doctor.

There is no proven efficacious intervention for preventing unnecessary cesarean sections. A promising approach, now being tested in six Latin American countries, is to request a second medical opinion before a cesarean section.^21^

An additional concern is that cesarean section rates are lower for high-risk than for low-risk women,^19^ since the latter are often private patients. Therefore, even in the presence of high rates, women at greatest need may still fail to get the cesarean section they need.

Only two Brazilian studies have investigated the association between cesarean sections and neonatal mortality. In Pelotas (South), babies delivered through a cesarean section had 1.6 times greater risk of death than those delivered vaginally.^16^ On the other hand, in Maringá (also in the South), normal delivery was associated with a 2.2-fold increase in death due to perinatal causes.^23^ In neither study were socioeconomic variables or level of gestational risk controlled for. Also, elective cesarean sections were not separated from non-elective; this may bias the results since delivery complications may often lead to a cesarean section.

No studies were found on the effect of neonatal care on mortality. However, there is indirect evidence for such an effect. Comparison of two birth cohorts in Pelotas (South region) showed that low birth weight rates increased slightly between 1982 and 1993,^24^ but nevertheless neonatal mortality rates were reduced by about 30%, from 20.1 to 14.3. In 1982, there were no neonatal intensive care units in the city, but by 1993 the three major maternity hospitals had such units. For the state of Rio Grande do Sul, low birth weight rates remained stable between 1980 and 1992 but neonatal mortality fell by 45%.^25^ During this period, there was a large expansion in neonatal intensive care in the state.

Given the small number of studies on mortality levels associated with place of delivery, cesarean sections and neonatal care services, and also due to the difficulties in interpreting these studies, it is not possible to carry out a formal simulation of the likely impact of these factors on mortality. However, accumulated knowledge suggests that increasing the rate of hospital deliveries, avoiding unnecessary cesarean sections, and improving neonatal care, will have a beneficial effect on neonatal mortality. There is considerable room for improvement in all of these areas in Brazil.

This conclusion is reinforced by the fact that perinatal and neonatal mortality levels in Brazil are quite high relative to the frequency of low birth weight. Studies from Pelotas (South)^26^ and Fortaleza (Northeast)^27^ show exceedingly high perinatal mortality among babies with a normal birth weight, who were growing well *in utero* and probably died due to poor delivery care. Also relevant is the observation that in developed countries about 6% of infants are of low birth weight, and yet infant mortality is around 6 per thousand, or about seven times lower than in Brazil.^1^

Interventions to improve delivery care include the training of traditional birth attendants (TBA's) in rural areas^28^ but a functional referral system is essential since most complications cannot be dealt with appropriately by these workers.^29^ Since over 90% of all births take place in a hospital, training of staff and improving equipment and supplies is a priority area.

*Preventing low birth weight.* Low birth weight (LBW) is defined as a birth weight < 2500 g. It is determined by two separate processes: the duration of gestation and the rate of fetal growth. Thus, a fetus or newborn infant can have LBW either because he/she is born early (preterm birth) or is born small for his/her gestational age (small for date), and the most important reason for it is intrauterine growth restriction (IUGR). In Brazil, about half of LBW infants are preterm and half are small for date.^14,30^

A recent review^31^ showed that the main causes of preterm birth include genital infections, multiple births, maternal hypertension, low pre-pregnancy body mass index, uterine dysfunction and maternal heavy work. On the other hand, the leading causes of IUGR are low energy in-take during pregnancy, ethnic factors, low pre-pregnancy body mass index, short stature and cigarette smoking. Therefore, low birth weight is a combination of different conditions that require distinct interventions. Appropriate antenatal care will address most of these conditions, but the nutritional status of mothers before and during pregnancy also has an impact.

Table 3 shows the available information on LBW according to two sources: the 1996 DHS survey (where mothers were asked to recall the birth weights of children born in the last five years) and SINASC. Both sources provide remarkably good agreement - the maximum difference is by two percent points for the Center-West. Reported birth weights show good agreement with actual birth weights,^32^ but mothers were unable to recall the birth weight for one in five children in the Northeast, and one in seven in North. This was mostly due to home deliveries in which birth weight is not routinely measured. Since home delivered babies are more likely to belong to poor, rural families, rates of LBW for the North and Northeast may be underestimated.

However, even studies from public sector hospitals in the Northeastern state capitals -which cater to the urban poor - show relatively low rates of LBW: 7.4% in Fortaleza^27^ and 7.6% in Natal.^14^ Two studies from rural areas in Ceará state also found low LBW rates for children examined by traditional birth attendants: 5.0%^28^ and 5.5%^13^, although the weighing equipment was not standardized. The PACS - Programa Nacional de Agentes Comunitários de Saúde (National Program of Community Health Workers) keeps a large number of low-income urban and rural communities under permanent surveil-lance. In 140 municipalities of Ceará state (Northeast), the mean LBW prevalence in 1994-6 was 5.1%, with a range from 0.1 to 20.7% and a standard deviation of 4.5 percentage points.^33^ Taking a worst case scenario, we assumed a 15% LBW prevalence when mothers were unable to provide birth weight information in the DHS (Table 3). Even after this correction, regional differences were remarkably small.

There are several Brazilian studies providing information on relative risks of infant mortality associated with LBW. All studies show increased risks of mortality for LBW babies. The three studies having infant mortality as the outcome^13,34,35^ show relative risks of 11.0, 9.7 and 6.1. Studies that separated preterm from growth-restricted LBW babies have shown that infant mortality among the former is at least twice as high as for the latter.^14,30,35^

Preventing low birth weight, when baseline levels are not very high, as is the case for Brazil, is not an easy task. One promising intervention is detection and treatment of asymptomatic bacteruria in pregnancy; a recent meta-analysis^36^ showed a 40% reduction in preterm deliveries among women with bacteruria but its author advised caution in the interpretation of these results due to methodological deficiencies in the studies. Detection and treatment of bacteruria is a standard component of ANC recommendations in Brazil.

Even in Southern Brazil where health care is among the best in the country and mortality is lowest, LBW rates have been stable or even increasing during the last couple of decades.^24,25^ The national prevalence estimate of 8.5% is only slightly higher than the current 6.0% prevalence in developed countries and considerably lower than the global estimate of 18% for less developed countries.^1^ Based on a relative risk of 9.0 for infant mortality among LBW babies, a reduction from 8.5% to 7.0% would prevent 8.3% of all infant deaths.

Two specific approaches for improving LBW - improving maternal nutrition and reducing smoking during pregnancy - are discussed below.

*Improving maternal nutrition.* Improving women's nutrition may help prevent LBW, since both preterm deliveries and intra-uterine growth restriction are more common among malnourished women. Two nutritional indicators are often used for mothers, BMI (body mass index, calculated by dividing weight in kg by the square of height in cm), and height. BMI's below 18.5 kg/m^2^ reflect undernutrition, while those over 25 reflect overweight and are associated with an increased risk of mortality due to chronic diseases.^37^

Table 3 shows that mothers are tallest in the South and Southeast and shortest in the North and Northeast. A similar pattern was observed for body mass index, but women from the Center-West also presented lower levels. Few women had a BMI under 18.5: the highest prevalence was in the Center-West (8.1%), and the lowest in the South (2.7%).

Brazilian studies confirm the well-known association between maternal nutrition and birth weight.^31,38,39^ Two studies showed an association between anthropometry and early neonatal mortality. In Pelotas (South)^16^ maternal pre-pregnancy weight was associated with mortality in the unadjusted analysis; maternal height was initially associated but this was no longer significant after controlling for family income. In Natal (Northeast)^14^ babies whose mothers weighted less than 50 kg were 1.4 times (1.0-1.8) more likely to die. The small number of studies and the format in which their results are presented do not allow impact estimates to be derived.

In any case, the feasibility of improving anthropometric indicators in large populations is limited. Adult height is largely determined in early life, so that current interventions will take a long time to reach an effect. Also, the secular trend in growth is present in all Brazilian regions and, although it may have slowed down recently, women's heights are continuing to increase over time.^40^

Screening high-risk mothers - for example, those with low BMI or short stature - is a possible alternative. Unfortunately, however, available data suggest that the risk approach is not very effective,^41^ since screening based on these anthropometric has both low sensitivity and low specificity.^41,42^ In other words, screening both fails to pick up many women with adverse pregnancy outcomes and identifies many women as being at risk who have normal pregnancy outcomes. Also discouraging is the low efficacy of the interventions themselves. Maternal energy supplementation has been shown to result in only a modest increase in energy intake, since much of the supplement appears to displace the normal diet.^43^ The consequence has little impact on fetal growth and no clear impact on the duration of gestation. Thus, many women would have to be screened and treated to prevent a few cases of IUGR or preterm birth. The combination of the low sensitivity of anthropometric screening and low efficacy of the intervention indicates that such a risk approach would make only a small dent in the overall prevalence of adverse pregnancy outcomes, even in developing countries where they are quite common.^31,41^

A population approach - encouraging all mothers to eat more during pregnancy - is likely to increase obesity rates^31^ which are already rising in Brazil, particularly in the Northeast.^44^ The mean BMI levels in Table 3 are already quite close to the maximum recommended level of 25.

*Reducing maternal smoking.* In the 1989 National Health and Nutrition Survey (PNSN), 27.1% of women aged 15-49 years were smokers (special tabulations provided by the National Cancer Institute). No regional estimates are available. A review of the Brazilian literature^45^ showed that prevalences of smoking among pregnant women were of similar magnitude. This review confirmed the international literature^31^: there were clear effects of maternal smoking on low birth weight and intrauterine growth restriction; effects on preterm deliveries were not consistent, but tended to show that children of smokers were at a higher risk. Brazilian studies on smoking and mortality - mainly perinatal -showed relative risks ranging from 1.0 to 1.4.^45^ Three studies detected significant differences, while four found no differences.

An international meta-analysis of the efficacy of smoking cessation programs in pregnancy showed a 49% reduction in smoking, a 19% decrease in low birth weight and an 18% non-significant reduction in preterm birth, but no effect was detected for very low birth weight or perinatal mortality.^46^ Based on this literature, there is a possible effect of smoking on early mortality, but the increase in risk appears to be very small.

*Promoting family planning.* The Brazilian literature shows that three variables related to reproductive behaviors are associated with increased infant mortality: teenage pregnancies, short birth intervals and high parity.

Table 3 shows that mothers aged less than 20 years accounted for 18.1% of deliveries reported in the 1996 DHS. This proportion was highest in the North (23.5%) and Northeast (20.6%) and lowest in the South and Southeast (16.2% in each). Of even greater concern is the fact that teenage pregnancies are on the increase: in the 1986 DHS^8^, 10.5% of women aged 15-19 years had already had a live birth whereas ten years later this proportion had increased to 14.3%.^6^

In the 1996 DHS, indirectly-estimated infant mortality for mothers aged under 20 years was 30% higher than for those aged 20-29 (the lowest risk category)^6^. These data were not adjusted for socioeconomic factors, and such adjustment is important because many teenage mothers come from poor families and poverty may confound the results. Results of four studies that took into account socioeconomic confounders^13,16,17,47^ showed no evidence that children of teenage mothers have an increased risk of mortality, a finding which is in agreement with a recent international review.^48^ Infants born to very young mothers (say, under 15 years) may be at higher risk but they are relatively few compared to those of mothers aged 18-20 years, who show no greater risk than women from other age groups.

Results on birth intervals are more compelling. Nationally, 29.2% of all births take place after a short (< 24 months) birth interval, but this proportion ranges from 18.1% in the South to 37.7% in the Northeast (Table 3). This shows a substantial reduction from the 46.3% national rate observed in the 1986 DHS.

In the 1996 DHS, short intervals were associated with a 2.2 times higher risk of under-five mortality,^6^ but these results were not adjusted for socioeconomic confounders which may exaggerate the effect. Three Brazilian studies that adjusted for socioeconomic confounding show increased mortality risks for children born after a short birth interval although not all differences were statistically significant.^47,49,50^

It is difficult to pool these results into a single estimate because different cutoff points were used, but it appears that birth intervals under 24 months are roughly associated with a 1.5 to 2-times greater risk of infant death. Using a pooled risk of 1.8, reducing by half the proportion of birth intervals under 24 months would lead to a 9.5% reduction in the number of infant deaths.

The role of high parity, or birth order, as a risk factor for infant mortality was also examined. The 1996 DHS tabulations do not provide a breakdown of birth order by region, but Table 3 shows that the total fertility rate (mean number of children had by women who completed their reproductive life) is highest in the Northeast (3.1) and lowest in the Southeast (2.2).

In the 1996 DHS,^6^ under-five mortality was 60% higher (relative risk of 1.6) among children with a birth order of three or more; these results are not adjusted for socioeconomic status, and there was no significance testing. All four Brazilian studies with confounder-adjusted relative risks tended to show some increase in risk for children of high birth order, but none were statistically significant.^13,15,17,49^

The velocity of fertility decline in Brazil is one of the fastest in the world: the total fertility rate decreased from 6.3 in 1970 to 2.6 in 1995.^51^

This trends continues, and the country is likely to reach replacement fertility by the year 2015.^52^ In the 1996 DHS, 76.7% of women in a steady relationship were using contraceptive methods^6^ (Table 3).

### Potential interventions against mortality due to congenital malformations

As other causes of death are being controlled, congenital malformations are playing a larger proportionate role. They are now the second cause of infant mortality in the country, accounting for 11.2% of these deaths. In 1997, cardiovascular defects accounted for 39.4% of all infant malformation deaths, and the nervous system for 18.8%. Table 1 shows that regional variability is minimal, since these deaths are extremely difficult to prevent in all parts of the world.

Strategies to reduce death rates due to congenital malformations include: ensuring an adequate folate intake around the time of conception, either by using vitamin supplements^53^ or through food fortification;^54^ avoidance of alcohol and of any drugs or medicines during pregnancy; treatment of diabetes before conception and continued control during pregnancy; and fetal screening and selective abortions.

Diagnosis and treatment of diabetes, as well as advice on alcohol and drugs, should be part of high-quality antenatal care. Investments in ANC aiming at reducing maternal and perinatal-cause deaths should also contribute to a reduction in malformations. Folate fortification or supplementation is still not being considered as a Public Health measure. Inducing abortions because of malformed fetuses is illegal in Brazil.

There are no data on the coverage of these interventions at regional level. Each one of these measures addresses one or a few specific types of malformations that only account for a small proportion of all birth defects. Therefore, neither of them is likely to have an important impact on infant mortality.

### Prioritizing interventions against perinatal causes

A primary strategy for reducing mortality due to perinatal causes is to improve equity in regional terms. Mortality rates are consistently higher in the Northeast and North relative to the other three regions. Of particular concern are mortality levels in rural areas. The 1996 DHS estimates that the indirect IMR was 55% higher in rural than in urban areas. Intervention coverage levels are also systematically lower in these areas.^6^

While health statistic levels in the Northeast are the least favorable, for most of them, rural areas in the North region are not covered by surveys, and few if any deaths in these remote areas are registered. Since almost 40% of the North's population is rural - the highest proportion in the country - and mortality is higher in rural than in urban areas, true levels of health in the North must be at least as poor as in the Northeast. Both regions, therefore, should deserve the highest priority in order to improve equality.

The following sections address possible interventions against mortality due to perinatal causes and malformations. Table 4 summarizes these interventions and – whenever possible – estimates their potential impact on overall infant mortality.

**Table 4:** Potential impact and feasibility of interventions against infant mortality due to perinatal causes in Brazil

Intervention	Assumptions	Infant deaths likely to be prevented (%)	Current levels and trends in risk factor or intervention	Feasibility of intervention
Improving antenatal care	Reducing by half the current proportion (31.9%) of women with less than 5 attendances	9.1%	The mean number of attendances is adequate but many high-risk women have too few attendances	Medium. Would require public education as well as improving accessibility.
	Improving the quality of antenatal care	Potentially large	Little information is available but average quality appears to be poor	Medium-high. Would require training health workers and providing drugs and equipment
Improving delivery care	Increasing the proportion of deliveries by a trained professional	Low to medium	86% of all births are already assisted by a doctor or nurse, and this rate has been increasing steadily.	Medium-low. Involves accessibility problems in rural areas as well as training of staff.
	Improving the quality of delivery care in hospitals	Potentially very large	The high perinatal mortality of babies with appropriate birth weight suggests that quality is poor	Medium-high. Would require training and providing equipment.
	Avoiding unnecessary cesarean sections	Low	36% of all deliveries are by a cesarean section, and this is still rising. But there is no strong effect on infant mortality.	Low. Social, cultural and economic factors are responsible for the high rate.
Improving birth weight	Current rate of 8.5% could be lowered to 7.0% (developed countries rate is currently 6.0%)	8.3%	Current levels already low. Time trends stable in the South and Southeast; no information for other regions	Low. Interventions against low birth weight have limited efficacy unless adult malnutrition is highly prevalent.
Improving maternal nutrition	Improving maternal body mass index and improving maternal height.	Low	Secular trend in growth is present in all regions and women tend to be larger. Improving height requires intervention to start in childhood.	Low. Nearly all interventions are ineffective and may lead to obesity.
Reducing maternal smoking	Reducing smoking from the current rate of 27%.	Low or none	27% of women smoke and this rate is not being reduced. No clear association with infant mortality.	Medium-high according to the international experience.
Promoting family planning	Preventing teenage pregnancies	Low or none	14% of women aged 15-19 are or have been pregnant; the trend is rising. But there is no clear association with infant mortality.	High, but there may be limited need for extra investment since Brazil is experiencing one of the most dramatic fertility declines in the world. Contraceptive use rates are high and still rising.
	Reducing by 50% the proportion of short birth intervals	9.5%	Proportion of birth intervals <24 months fell from 46% to 29% from 1986-96.	As above
	Reducing the proportion of high-parity women	Low or none	Family sizes are dropping rapidly. There is no clear association with infant mortality.	As above
Preventing malformations	Improving antenatal care quality to prevent and treat risk factors for malformations	Low	Current quality of antenatal care appears to be low	Medium-high for improving ANC: requires training health staff and providing access to laboratory facilities. But malformations have multiple etiologies, each one contributing to a small number of deaths

### Need to prioritize delivery and antenatal care

According to Table 4, antenatal and delivery care are potentially the highest impact measures for further reduction of infant mortality. Perinatal-cause mortality is particularly high in the Northeast and North, and interventions against perinatal causes are expected to reduce infant mortality rates.

It is hard to make a precise estimate of the potential contribution of improving standards of delivery and antenatal care, but the high levels of perinatal mortality given the moderate rates of low birth weight point out to serious shortcomings in delivery care.

Interventions in this area include ensuring at least five antenatal attendances to every woman, ensuring that all births are attended by a skilled health worker (such as a doctor or a nurse), and particularly retraining and supervising health staff in antenatal and delivery services. Nationally, there is an urgent need to improve quality of care in hospitals, where almost 90% of births now take place. In rural areas, traditional birth attendants will have to be trained to refer women with delivery complications.

### Need for birth spacing interventions

Reducing by half the proportion of birth intervals that are under 24 months can theoretically prevent 9.5% of infant deaths. Although family planning is now used by three quarters of women in stable relationships,^6^ and this rate is increasing, the lower contraceptive use rates in the Northeast (68%) show room for improvement (Table 3).

### Need to prevent LBW

Reducing LBW prevalence from 8.5% to 7.0% will theoretically lead to an 8.3% decrease in infant mortality. However, baseline levels are already low and available interventions have limited efficacy, making this a difficult goal to achieve.

Since LBW babies are at a 10-fold greater risk of mortality, a more promising strategy may be target these infants for close follow-up activities by health services, a program that is already being implemented in some states. Further information on the effectiveness of these programs is required before they can be endorsed for wider use.

For the time being, no additional investments in LBW prevention - other than those resulting from improving antenatal care and preventing smoking during pregnancy, as discussed above - are recommended.

## CONCLUSION

Current levels of infant mortality are unacceptably high in the view of the economic potential of Brazil. Regional differences within the country are exceedingly large and ethically inadmissible.

Infant mortality levels have been falling since the 1970's but there is still scope for major reductions since current levels are about ten times higher than in the world's most developed countries.^1^ Almost 60% of the infant deaths in Brazil are due to perinatal causes, and therefore the efforts to reduce infant mortality should contain a strong component of health actions aiming at preventing early neonatal deaths.
